# Bisphenol a Exposure, DNA Methylation, and Asthma in Children

**DOI:** 10.3390/ijerph17010298

**Published:** 2020-01-01

**Authors:** Chia-Feng Yang, Wilfried J. J. Karmaus, Chen-Chang Yang, Mei-Lien Chen, I-Jen Wang

**Affiliations:** 1 Department of Pediatrics, Taipei Veterans General Hospital, Taipei 112, Taiwan; pum_chia@yahoo.com.tw; 2 Institute of Environmental and Occupational Health Sciences, National Yang-Ming University, Taipei 112, Taiwan; ccyang2@ym.edu.tw (C.-C.Y.); mlchen@ym.edu.tw (M.-L.C.); 3 Division of Epidemiology, Biostatistics, and Environmental Health, School of Public Health, University of Memphis, Memphis, TN 38152, USA; karmaus1@memphis.edu; 4 Department of Pediatrics, Taipei Hospital, Ministry of Health and Welfare, Taipei 242, Taiwan; 5 School of Medicine, National Yang-Ming University, Taipei 112, Taiwan; 6 College of Public Health, China Medical University, Taichung 400-439, Taiwan; 7 National Institutes of Environmental Health Sciences, National Health Research Institutes, Miaoli 35053,Taiwan

**Keywords:** bisphenol A, DNA methylation, asthma

## Abstract

Epidemiological studies have reported the relationship between bisphenol A (BPA) exposure and increased prevalence of asthma, but the mechanisms remain unclear. Here, we investigated whether BPA exposure and DNA methylation related to asthma in children. We collected urinary and blood samples from 228 children (Childhood Environment and Allergic Diseases Study cohort) aged 3 years. Thirty-three candidate genes potentially interacting with BPA exposure were selected from a toxicogenomics database. DNA methylation was measured in 22 blood samples with top-high and bottom-low exposures of BPA. Candidate genes with differential methylation levels were validated by qPCR and promoter associated CpG islands have been investigated. Correlations between the methylation percentage and BPA exposure and asthma were analyzed. According to our findings, *MAPK1* showed differential methylation and was further investigated in 228 children. Adjusting for confounders, urinary BPA glucuronide (BPAG) level inversely correlated with *MAPK1* promoter methylation (β = −0.539, *p* = 0.010). For the logistic regression analysis, *MAPK1* methylation status was dichotomized into higher methylated and lower methylated groups with cut off continuous variable of median of promoter methylation percentage (50%) while performing the analysis. *MAPK1* methylation was lower in children with asthma than in children without asthma (mean ± SD; 69.82 ± 5.88% vs. 79.82 ± 5.56%) (*p* = 0.001). Mediation analysis suggested that *MAPK1* methylation acts as a mediation variable between BPA exposure and asthma. The mechanism of BPA exposure on childhood asthma might, therefore, be through the alteration of *MAPK1* methylation. The mechanism of BPA exposure on childhood asthma might, therefore, be through the alteration of *MAPK1* methylation.

## 1. Introduction

The use of harmful chemicals has been increasing in modern society. There has also been a simultaneous increase in the number of asthma and other allergic disorders in children. Global Initiative of Asthma reported in 2004 that as many as 300 million people of all ages have asthma, and the estimated number might be further increased in the future [1].

In Taiwan, the prevalence of children affected by asthma was around 12%; asthma is one of the most important health issues in children in Taiwan and worldwide [2,3]. Our previous studies and those of others have shown that children with asthma have difficulty in learning due to hyperactive and impulsive behaviors that are secondary consequences of the illness [4,5], resulting in a considerable burden on public health.

BPA is an endocrine-disrupting chemical being increasingly used in modern society. Hence, health concerns regarding BPA use should not be ignored [6]. Some epidemiological studies have presented the association between BPA exposure and a higher prevalence of asthma [7]. Pulmonary pathological data further support that BPA might aggravate asthma through adjuvant effects [8]. In our previous study, we discovered that BPA exposure was related to high IgE levels in children [9]. We also discovered that the urine BPA metabolite level was significantly higher in Taiwanese children than in those of other countries [9,10]. BPA has become serious public health problem globally and been described the various epigenetic mechanisms, like DNA methylation, histone modifications and non-coding RNAs, then affecting gene expression [11,12,13]. Furthermore, we found that exposure to this harmful chemical affected DNA methylation, which influenced the prevalence of allergic disorders in children [14]. Therefore, epigenetic variations caused by environmental factors might be associated with the development of asthma.

Multiple exposure routes including oral, dermal, and airway routes contribute to the total intracellular BPA concentration in children [15,16]. The increasing health concern was attributed to the continuous low-level exposure to BPA in children [15,16,17]. BPA is not bio-accumulative, but continuous exposure from many sources makes it as harmful as a bio-accumulative compound [18]. According to a recent report, BPA has a longer than expected half-life [16]. Some animal studies have reported that several endocrine-disrupting environmental chemicals can modify epigenetic marks [19]. Several studies have focused on the epigenetic modifications caused by environmental exposure to BPA, but most of them have not been directly linked to a clinical endpoint [20,21]. Further, there is limited information available from large-scale data regarding children exposed to BPA based on clinical specimens. In this study, we investigated the effects of BPA exposure on epigenetic modification and established a correlation between healthy and asthmatic children. This study focused on whether exposure to BPA induces aberrant DNA methylation of specific genes related to childhood asthma in children aged 3 years.

## 2. Materials and Methods

### 2.1. Study Population

A total of 228 3-years-old children for whom urine and blood specimens were available from the Childhood Environment and Allergic Diseases Study (CEAS) cohort were included in this study [14,22]. Full enrollment was completed by monitoring BPA exposure, analyzing the urinary BPA glucuronide (BPAG) level, and sampling of blood. The guardians were interviewed at pediatric clinics to obtain information regarding gender, prematurity, maternal age, history of atopy, educational level, breastfeeding or formula feeding, and exposure to environmental tobacco smoke (ETS) exposure, family income and asthma history of the children. Informed consent was obtained and the study was approved by the Institutional Review Board of the Taipei Hospital (IRB No. TH-IRB-09-04).

### 2.2. Determination of Cases

The International Study of Asthma and Allergies in Childhood (ISAAC) questionnaire was provided to the guardians of the children included in the study [3]. Asthma patients were defined as “physician-diagnosed asthma.” Pediatric allergists accomplished a standardized history examination of participants and the guardians were asked to report whether their child suffered from wheezing or used asthma medications through a questionnaire using three criteria: (i) recurrence of at least two of the three symptoms: cough, wheeze, and shortness of breath within the past 12 months without having a cold, (ii) doctor’s diagnosis of asthma with ongoing treatment, and (iii) response to treatment with β2-agonists or inhaled corticosteroids [23,24]. Those who were not able to answer the questions, had multiple gestation, and did not follow up regularly were excluded from the study.

### 2.3. Laboratory Method

#### 2.3.1. Exposure Monitoring

The first mid-stream urine in the morning was collected from children and stored at −20 °C before analysis. The specimen was analyzed by solid phase extraction method. We measured the concentrations of urinary BPA glucuronide (BPAG) of children aged 3 years as an indicator of exposure [9,25]. BPAG was determined by the method of ultra-performance liquid chromatography and tandem mass spectrometry (UPLC-MS/MS) using isotope-dilution techniques [9,25,26]. The limit of detection (LOD) was 1.61 ng/mL [9] and for a reported value less than the LOD, one-half the limit of detection was assigned. All experiments were done in duplicates for data acquisition. Urine creatinine levels were detected by enzymatic assay (Cayman Chemical, Ann Arbor, MI, USA) and urinary BPAG was adjusted for urine creatinine levels.

#### 2.3.2. Selection of Candidate Genes by Methylation-Dependent Fragment Separation (MDFS) 

**Figure 1:**
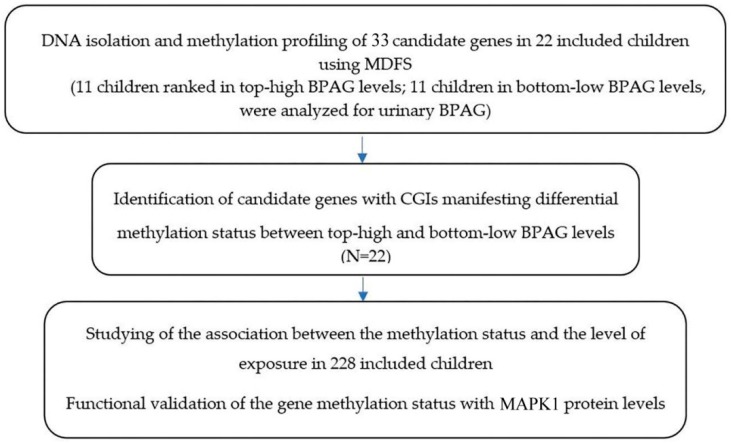
Flow chart describing this study.

From the toxicogenomics database, we selected 33 human candidate genes having CpG islands, which are known to interact with BPA [6]. These genes included *ESR1*, *ESR2*, *AR*, *PGR*, *ESRRG*, *THRB*, *CjYP1A1*, *CYP19A1*, *VEGFA*, *MAPK1*, *MAPK3*, *STAT3*, *LIF*, *NR1I2*, *TFF1*, *TNFα*, *IL-4*, *S100G*, *LHB*, *GH1*, *NR4A1*, *HOXA10*, *CYP11A1*, *CYP17A1*, *PRL*, *STAR*, *IGF1*, *NCOA1*, *P4HB*, *DDIT3*, *FOS*, *HSP90AA1*, and *THRA*. The candidate genes were chosen based on the following criteria: (i) those genes that interacted with BPA, and (ii) those that were known to be associated with asthma. Figure 1 shows the study flow chart of the selection of candidate genes associated with BPA exposure.

Initially, we investigated DNA samples of 11 children who were ranked as having the top-high BPAG exposure levels and 11 age- and gender-matched children who were ranked as having the bottom-low BPAG exposure levels. The 22 blood samples were screened by utilizing methylation-dependent fragment separation (MDFS) to assess the differences of methylation between top-high and bottom-low BPA exposure groups. MethPrimer software was used to recognize CpG islands (CGIs) of selected candidate genes and design primers for polymerase chain reaction (PCR) by EpiTect Methl II qPCR.

#### 2.3.3. Analysis of the Methylation Status by Quantitative PCR (qPCR) and Pyrosequencing in a Cohort of 228 Children

The genomic DNA were derived from peripheral blood of all subjects and extracted by DNeasy Blood and Tissue Kit (Cat No./ID: 69506, QIAGEN, Inc., Valencia, CA, USA). Candidate genes with differential methylation level in two exposure groups (top-high and bottom-low BPA levels) were identified through a screen with MDFS and further validated using a qPCR method or pyrosequencing in all samples. The qPCR method was based on the detection of input DNA after digestion with a methylation-dependent restriction enzyme that cleaves unmethylated and methylated DNA [27,28]. The bisulfate conversion of DNA was treated with EpiTect^®^ Plus Bisulfite Conversion K it (#59124, QIAGEN, Inc.). The bisulfate conversion efficiency was monitored by reference DNA set (included unmethylated DNA and methylated DNA) (#59695, QIAGEN, Inc.). Restriction digestion was performed using the EpiTect Methyl II DNA Restriction Kit (SAB# 335452). Following digestion, the remaining DNA in each individual enzyme reaction was quantified by ViiA7 real-time PCR instrument. The assay primers were designed and synthesized by QIAGEN SABioscience (QIAGEN, Inc.). The relative fractions of methylated and unmethylated DNA are subsequently determined by comparing the amount in each digest with that of a mock (no enzymes added) digest using a ΔCT method.

Since there was no commercial kit for qPCR-based DNA methylation of the *IL-4* gene and *MAPK1*, we validated DNA methylation of *IL-4* and *MAPK1* result by a customed designed pyrosequencing method [29,30]. The PCR reaction was conducted using PyroMark PCR Kit (#978703, QIAGEN, Inc.) with specific primers for IL-4 gene as below: Forward 5’-GTTGATTGGTTTTAAGTGATTGATAATT-3’ and backward 5’-Biotinylated ATACCCAAATA AATACTCACCTTTCACT-3’; *MAPK1* gene: Forward 5’-TGAATGTATTGTGAATGTATGT GATTGT-3’ and backward 5’-Biotinylated GAGAGTTGAAGAGTTGAT ATGTTATTTGG-3’. The PCR program was performed by the Veriti Thermo Cycler (# 4375786, Life Technologies, Carlsbad, CA, USA) and then the PCR products were separated into single strands using streptavidin-coated beads. The pyrosequencing was applied with specific sequencing primer (*IL-4*: 5’-TTTTTGTTTTT TTTGTTAGTATGT-3’ and *MAPK1*: 5’-TTTTTAGTTAA TGTTGTTGTAGTG-3’) using PyroMark Gold Q24 Reagents (#970802, QIAGEN, Inc.) which was designed for CpG methylation analysis. The sequence signals were generated by PyroMark Q24 instrument and analyzed the sequence peak signal intensity by PyroMark Q24 software v2.0.6. The association between methylation status of the candidate gene, exposure levels, and asthma were further analyzed.

#### 2.3.4. Analysis of Plasma Level of MAPK1 Protein

For the quantitative measurements of MAPK1 (ERK1/2), an ELISA kit was used on lysed human leukocytes as described (ERK1/2 SimpleStep ELISA Kit, ab176641, Abcam, Cambridge, UK) [27]. Briefly, fifty microliters of human leukocytes lysates in Cell Extraction Buffer PTR were added to the wells, followed by the antibody mix (50 microliters). After incubation for an hour at room temperature, the wells are washed to remove unbound material by buffer PT. One-hundred microliters of TMB substrate was added to each well and incubate for 15 min in the dark. After incubation, the reaction was stopped by addition of one-hundred microliters of Stop Solution, and the intensity was measured at 450 nm.

### 2.4. Statistical Analysis

Variables with skewed distributions were going to be log (Ln)-transformed then taking analyses in the next step. All data after log-transformed in this study presented a normal distribution, and no significant outliers were found. Associations between urine BPAG levels and the *MAPK1* promoter Met% were evaluated by linear regression. Association between BPAG level and asthma was analyzed by univariate and multivariate logistic regression. Independent t-tests were performed to assess differences of methylation percentage (Met%) in the promoter methylation percentages (Met%) of 33 human candidate genes which are known to interact with BPA genes identified by methylation-dependent fragment separation (*N* = 22) [6]. To analyze the association of *MAPK1* 5′CGI methylation status with asthma in CEAS cohort, we first compared the mean of methylation levels. A logistic regression analysis was performed to evaluate the association of methylation status of target genes with asthma. For the logistic regression analysis, we dichotomized the methylation into higher and lower groups. *MAPK1* methylation status was dichotomized into higher methylated and lower methylated groups with cut off continous variable of median of promoter methylation percentage (50%) while performing the analysis. Selection of the confounders have been kept in the exploratory model, which was based on the literatures and the standard statistical procedures, avoiding a change of more than 10% in the point estimate of the exposure. All tests assumed a two-sided alternative hypothesis and provide estimates of effects with exact *p*-values. All analyses were conducted using SAS software version 9.1 (SAS Institute, Cary, NC, USA).

## 3. Results

### 3.1. Selection of the Most Relevant Candidate Genes

After screening 33 candidate genes in 22 included children by MDFS, we observed marginal differences methylation status of the promoter region for four genes (*AR*, *TNFα*, *IL-4*, and *MAPK1*) between the top-high and bottom-low BPA exposure groups, which had marginal significances methylation identified by methylation-dependent fragment separation. All the required information was available for 453 children aged 3 years. Table 1 presents the basic demographic data of the study population. We excluded children who discontinued follow-up (*n* = 84) and whose blood or urine data (*n* = 75) or outcome data (*n* = 66) were not available, and the final number of the sample cohort studied was 228. The basic demographics of the study population showed no significant differences between those who discontinued to follow-up and those who completed follow-up (Table 1).

These four genes were selected for further investigation (Table 2) A review of the relevant literature showed that these genes were related to the development and maintenance of the male sexual phenotype (androgen receptor; *AR*, Gene ID: 367); stimulation of the acute phase proinflammatory cytokine and regulator of immune cells (tumor necrosis factor alpha; *TNFα*, Gene ID: 7124); activation of B-cells; promotion of T-cell proliferation; induction of B cell switching to IgE (interleukin 4; *IL-4*, Gene ID: 16189); production of pro-inflammatory mediators; mediation of cell growth, adhesion, survival, and differentiation; and regulation of meiosis, mitosis, and post-mitotic functions (mitogen-activated protein kinase1; *MAPK1*, Gene ID: 5594) (Table 2). The percentage of promoter methylation (Met %), as measured by qPCR, of these candidate genes, tested for 22 samples, is presented in Table 3. Only *MAPK1* showed a differential methylation status between the top-high and bottom-low exposure groups after validation by qPCR (low vs. high exposure: 79.82 ± 5.56 vs. 69.82 ± 5.88, *p* = 0.001).

### 3.2. Relationship of MAPK1 5′CGI Methylation Status with BPA Exposure and Asthma

After adjusting for confounders, urinary BPAG levels maintained a significant negative regression coefficient for the *MAPK1* 5’CGI promoter methylation percentage (Met %) in the initial cohort (β = 0.83, *p* = 0.022) (Table 4). Table 5 presents the association of the *MAPK1* 5’CGI methylation status with asthma. For the logistic regression analysis, we dichotomized the methylation into higher and lower groups. *MAPK1* methylation status was dichotomized into higher methylated and lower methylated groups with cut off continous variable of median of promoter methylation percentage (50%) while performing the analysis. Lower methylation of the *MAPK1* 5’CGIs was found to be positively associated with asthma compared to higher methylation of *MAPK1* 5’CGIs (adjusted OR = 2.33, 95% CI = 1.01–5.39, *p* = 0.020).

To investigate whether the *MAPK1* 5′CGI methylation status affects its protein expression, we also determined the plasma MAPK1 protein level. The mean level ± SD was 901.78 ± 11.51 pg. We found that *MAPK1* methylation (Met %) was significantly inversely related to the MAPK1 protein level (β = −0.18, p = 0.041). Urinary BPAG levels were also significantly associated with asthma (adjusted OR = 1.52, 95% CI = 1.12–2.05), *p* < 0.05 (Table 6).

Table S1 presented the promoter methylation percentages (Met%) of 33 human candidate genes which are known to interact with BPA genes identified by methylation-dependent fragment separation (*N* = 22) [6].

## 4. Discussion

This study is the first large-scale assessment demonstrating a link between BPA exposure and asthma via epigenetic mechanisms in children. We demonstrated that a higher exposure to BPA is related to a lower DNA methylation of the *MAPK1* gene, which is associated with a higher risk of developing asthma. Furthermore, we showed that the protein level of *MAPK1* were inversely related to BPA exposure.

It has been shown that BPA is a harmful environmental chemical and its exposure may influence the human immune system [8,31]. BPA exposure might affect the immune system by releasing some pro-inflammatory mediators, including cysteinyl leukotriene, MAPK1, prostaglandin D2, and IL-13, which might be related to the development of asthma [32]. In addition, BPA exposure has been shown to affect many human chronic diseases, including diabetes, metabolic syndrome, reproductive disorders, cardiovascular diseases, respiratory diseases, and breast cancer [33]. Animal studies suggested that BPA exposure might reduce the levels of regulatory T cells, IL-10, and IFN-γ and increase the production of IL-4 and antigen-specific IgE [34,35]. Donohue et al. (2012) reported that urinary BPA levels at the ages of 3, 5, and 7 years were associated with childhood asthma between the ages of 5 and 12 years [7]. Pre-natal and post-natal BPA exposure has been reported to increase the odds of childhood asthma and allergic disorders [7,9,22,36,37,38,39,40]. Moreover, our previous study showed that higher BPA exposure was associated with increased serum IgE levels and might be related to the development of allergic disorders, particularly in children [9]. However, the mechanism underlying BPA-induced asthma remains unknown.

In our study, we found that BPA exposure was related to decreased methylation of *MAPK1* 5′CGI, which might be related to the development of asthma in children. Microtubule Affinity Regulating Kinase 1 (*MARK1*) is a protein-coding gene and a member of the MAPK signaling pathway. Among its functions are cytoskeletal signaling, energy metabolism, production of pro-inflammatory mediators, and cell growth and differentiation. Hung et al. demonstrated that circulating myeloid dendritic cells treated with two common environmental endocrine-disrupting chemicals, nonylphenol, and 4-octylphenol, increased the expression of tumor necrosis factor-α via the MAPK signaling pathway [41]. Another study showed that overexpression of a family of MAPKs in cells leads to hyperphosphorylation of microtubule-associated proteins and disruption of the microtubule array, resulting in morphological changes and cell death [42]. These findings indicate that exposure to endocrine disruptors can potentially alter the DNA methylation status of the *MARK1* gene.

Furthermore, prenatal exposure to BPA was reported to alter the methylation status of the genes related to reproductive processes in the animal [43] and human [38,39,44]. In addition, BPA could induce a dose-dependent activation of the pro-inflammatory cytokine MAPK1 and the formation of reactive oxygen species (ROS) in rat alveolar macrophages [42,45,46]. Therefore, BPA exposure might trigger airway macrophages to express *MAPK1*, mediated by alterations in DNA methylation, which then activates downstream signals to enhance inflammatory responses [47,48].

Higher BPA exposure was shown to lead to a decrease in *MAPK1* 5′CGI methylation in our study. Lower methylation of *MAPK1* 5′CGI is associated with increased gene expression, which in turn increases *MAPK1* expression at protein levels and triggers the development of allergic inflammation [49,50,51,52,53]. We demonstrated that the methylation of *MAPK1* 5′CGI is an intervening factor between BPA exposure and asthma. MAPK signaling pathways are known to be involved in airway inflammation and the regulation of immune cells, which are the hallmark features of asthma [49,50,53]. In addition, genetic variants of *MAPK1* might be involved in regulating cytokine levels in asthma patients, which might modulate the severity of asthma [54,55,56].

We initially found DNA methylation in the *AR* gene, which is related to BPA exposure. However, *AR* failed to display a significant difference in methylation after adjusting for potential confounders. In particular, sex differences in AR expression may also account for this finding.

There are a few limitations to our study. First, our study was confined to the use of the candidate genes approach. The candidate genes approach has limited accuracy due to the dependence on prior studies, which lead to an information bottleneck. Therefore, instead of only one candidate gene, we chose 33 candidate genes from a published toxicogenomics database to carry out our investigations. This approach provides biological plausibility to our study and is more cost-effective than the genome-wide approach. Second, RNA samples were not available for this study. High-quality RNA isolation from blood was technically difficult because of its limited cellular components. However, we assessed the relationship between the methylation and translation of genes via protein quantitation. Third, our analysis was based on a single morning urine sample. However, spot urine samples and 24-h urine samples have been reported to produce similar levels of daily BPA intake [57]. Even though BPA has a relatively short half-life, its continuous daily intake contributes to an exposure scenario that is similar to those of bioaccumulative compounds [58,59]. Additionally, if the measurements are not precisely and carefully performed, outcomes are nullified and the effects of exposure could be underestimated. Forth, the DNA-methylation information is derived from whole blood samples, which includes other cell types besides lymphocytes. The whole blood consists of many functionally distinct cell populations. The interpretation of DNA-methylation profiles from whole blood should be conducted with great caution, because the differences might be resulted from varying proportions of white blood cell types. In this study, we analyzed DNA-methylation status of 33 genes in whole blood DNA, and found methylation status of *MAPK1* gene was associated with BPA exposure. However, the FACS sorting approach might be needed to isolate neutrophils, B and T lymphocytes, monocytes, and other granulocytes to study which subpopulation of whole blood cells are affected.

The strengths of this study are the collection of clinical specimens with available clinical and environmental exposure data. The use of urinary BPAG level analyzed by UPLC-MS/MS provided a more direct measure of individual BPA exposure. Furthermore, asthma was confirmed by pediatric allergists using a questionnaire. Diagnosis of asthma by pediatric allergists through questionnaires is a gold standard method [60]. Therefore, the errors in the classification of the outcome could be minimized through these means. Another strength of our study is that we employed a two-step study: MDFS method was used to identify candidate gene methylation and was then followed by qPCR to confirm the methylation status of the candidate genes in a larger sample cohort of 228 children. This step-wise approach allowed us to identify the possible candidates in a cost-effective manner.

## 5. Conclusions

In conclusion, we found through this study, that the effect of BPA exposure on the development of asthma in children might be mediated through the alteration of DNA methylation. In particular, the *MAPK1* 5′CGI methylation status might act as an epigenetic biomarker for the induction of childhood asthma due to BPA exposure. Our findings contribute to a better understanding of the etiology of asthma and will aid the development of new strategies for the early prevention or therapeutic intervention of asthma. Further studies are needed to evaluate the long-term biological effects of BPA exposure in children.

## Figures and Tables

**Table 1:** Characteristics of the total population and the analyzed subsample.

Category	Subjects with Urine and Blood Specimens (*N* = 228)	Initial Cohort with Urine Specimens (*N* = 453)	*p*-Value
Gender (male) (%)	55.9	57.7	0.667
Prematurity <37week (%)	7.6	9.0	0.702
Maternal age ≥34 years (%)	24.0	17.8	0.126
Maternal history of atopy (%Yes)	40.7	35.1	0.408
Maternal education College (%)	25.4	30.8	0.370
Breast feeding (%Yes)	67.2	76.4	0.106
ETS exposure (%Yes)	59.5	46.2	0.066
Family income per year >1,500,000 (NT dollars) (%)	9.1	8.0	0.531
Asthma (% Yes)	24.6	26.9	0.507

ETS exposure: environmental tobacco smoke exposure; Chi squared tests was used to evaluate the variables in this table.

**Table 2:** The description and promoter methylation percentages (Met%) of four candidate genes with relatively differential methylation identified by methylation-dependent fragment separation (*N* = 22).

Gene (ID)	CpG Island Location	Gene Function	Map
	TSS Position	Promoter Methylation Percentage (Met%) (mean ± SD) upon Low and High BPA Exposure	
*AR* (367)	ChrX: 66763684–66764077	Development and maintenance of the male sexual phenotype, DNA-binding transcription factor that regulates gene expression	NM_000044 Genome Position: chX 66680589–66860844(+) 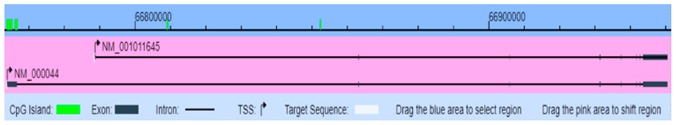
	66763873	Bottom-low vs. top-high exposure 37.76 ± 23.87 vs. 23.73 ± 18.33 *p* = 0.138	
*TNFα* (7124)	Chr6: 31543344–31544344	Pro-inflammatory cytokine- stimulates the acute phase reaction and airway inflammation and regulates immune cells	NM_000594 Genome Position: chr6 31651328–31654089(+) 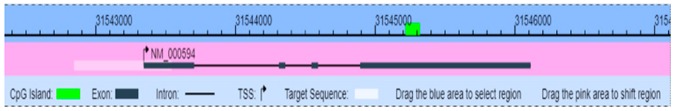
	31543350	Bottom-low vs. top-high exposure 42.15 ± 36.60 vs. 23.20 ± 22.37 *p* = 0.16	
*IL-4* (16189)	Chr5: 132035956–132036176	Activates B-cell and T-cell proliferation induces B-cell class switching to IgE	NM_000589 Genome Position: chr5 132037271–132046267(+) 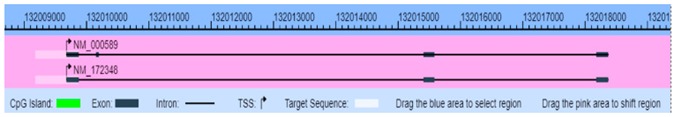
	132040541 Specific primers for IL-4 gene as below: Forward 5′-GTTGATTGGTTTTAAGTGATTGATAATT-3’ and backward 5′-Biotinylated ATACCCAAATAAATACTCACCTTTCACT-3’.	Bottom-low vs. top-high exposure 89.36 ± 7.65 vs. 85.73 ± 6.99 *p* = 0.26	
*MAPK1* (5594)	Chr22: 20443948–20551970	Mediates cell growth, adhesion, survival, and differentiation. Regulates meiosis, mitosis and postmitotic functions	NM_002745 Genome Position: chr22 2044394–20551970(-) 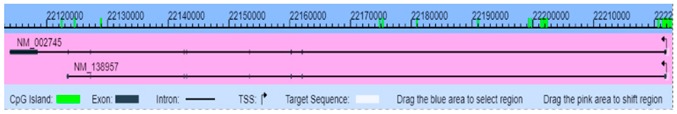
	20447613	Bottom-low vs. top-high exposure 79.82 ± 5.56 vs. 69.82 ± 5.88 *p* = 0.001	

Figure source: MethPrimer 2.0.

**Table 3:** Promoter methylation percentage (Met%) of four candidate genes with marginal significant differential methylation validated by quantitative PCR (*N* = 22).

Sample	BPAG Level (ng/mL)	*TNFα*	*p*-Value	*AR*	*p*-Value	*IL-4*	*p*-Value	*MAPK1*	*p*-Value
		Met%		Met%		Met% ^a^		Met%	
Bottom-Low Exposure									
L1	0.81	11.07	0.158	50.00	0.138	87.00	0.258	82.00	0.001
L2	0.81	85.22		33.86		88.00		87.00	
L3	0.81	73.88		6.57		71.00		81.00	
L4	0.81	14.41		50.00		88.00		75.00	
L5	6.55	67.60		8.36		100.00		86.00	
L6	6.55	14.38		79.64		89.00		79.00	
L7	6.55	11.04		27.36		91.00		75.00	
L8	6.55	94.64		52.27		89.00		76.00	
L9	6.55	76.97		65.62		88.00		84.00	
L10	6.55	6.85		28.80		92.00		84.00	
L11	6.55	7.57		12.91		100.00		69.00	
Top-High Exposure									
H1	86.55	34.31		51.32		81.00		71.00	
H2	96.26	5.59		4.57		84.00		66.00	
H3	99.03	10.16		11.95		71.00		73.00	
H4	115.60	6.14		50.43		91.00		74.00	
H5	137.20	23.49		14.76		91.00		69.00	
H6	143.20	22.76		13.19		87.00		72.00	
H7	147.40	21.59		36.34		90.00		69.00	
H8	155.10	84.09		4.78		82.00		77.00	
H9	239.50	28.02		13.66		80.00		60.00	
H10	260.50	10.39		14.32		90.00		60.00	
H11	392.00	8.63		45.65		96.00		77.00	

^a^ Met% is the percentage of methylated cytosine in the CGIs determined by pyrosequencing. Logistic regression analysis was used to evaluate the variables in this table.

**Table 4:** The association between the log-transformed BPAG level and standardized regression coefficient βeta for the *MAPK1* promoter methylation percentage (*N* = 228).

*MAPK1* Promoter Methylation Percentage (Met %)	Ln-BPAG	*p*-Value
Adjusted β ^a^	0.83	0.022 *

^a^ Adjustment for urine creatinine, white blood cell proportion, gender, age, maternal education, and environmental tobacco smoke exposure. BPAG: bisphenol A glucuronide. * *p* < 0.05. Linear regression analysis was used to evaluate the variables in this table.

**Table 5:** The association between the *MAPK1* 5′CGI methylation status and asthma (*N* = 228).

Association Between the MAPK1 5′CGI Methylation Status	Asthma (*N* = 56)	Non-Asthma (*N* = 172)	Subjects (*N* = 228)	OR (95% CIs)	Adjusted OR ^b^ (95% CIs)
Lower methylated *MARK1* 5′CGI ^a^	35 (62.5)	93 (46.5)	114 (50.0)	2.17 (1.27–3.68) *	2.33 (1.01–5.39) *
Higher methylated *MARK1* 5′CGI	21 (37.5)	107 (53.5)	114 (50.0)	1	1

^a^ The *MAPK1* methylation status was dichotomized into a lower and a higher methylated group with the median of promoter methylation percentage as the cut off value, ^b^ Adjustment for age, gender, prematurity, maternal history of atopy, and environmental tobacco smoke exposure. * *p* < 0.05. Logistic regression analysis was used to evaluate the variables in this table.

**Table 6:** The association of BPA exposure with asthma in children (*N* = 228).

BPA Levels	Ln-BPAG
Asthma Adjusted OR (95% CI) ^1^	1.52 (1.12–2.05) *

^1^ Adjustment for gender, age, prematurity, maternal history of atopy, maternal education, and environmental tobacco smoke exposure; * *p* < 0.05. Univariate and multivariate logistic regression were conducted in this table.

## Supplementary Materials

The following are available online at https://www.mdpi.com/1660-4601/17/1/298/s1, Table S1: The description and promoter methylation percentages (Met%) of 33 human candidate genes which are known to interact with BPA.
