# Supplementary material for: Bisphenol a Exposure, DNA Methylation, and Asthma in Children
Source: Int J Environ Res Public Health. 2020 Jan 1;17(1):298. doi: 10.3390/ijerph17010298 (PMC6981376; doi:10.3390/ijerph17010298)
Supplement: Supplementary file 1 [file ijerph-17-00298-s001.zip › ijerph-610965-supplementary.docx]

**Table S1.** The description and promoter methylation percentages (Met%) of 33 human candidate genes which are known to interact with BPA.

| **Gene**  **(ID)** | **CpG island Location** | **Gene Function** | **Map** |
| --- | --- | --- | --- |
|  | **TSS Position** | **Promoter Methylation Percentage (Met%)**  **(mean±SD) upon Low and High BPA Exposure** |  |
| *AR*  (367) | ChrX: 66763684 – 66764077 | Development and maintenance of the male sexual phenotype,  DNA-binding transcription factor that regulates gene expression | 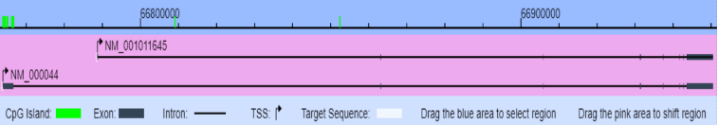  NM_000044  Genome Position: chX 66680589-66860844(+) |
|  | 66763873 | Bottom-low vs. top-high exposure  37.76±23.87 vs.23.73±18.33  p=0.138 |  |
| *TNFα*  (7124) | Chr6: 31543344 - 31544344 | Pro-inflammatory cytokine- stimulates the acute phase reaction and airway inflammation and regulates immune cells | 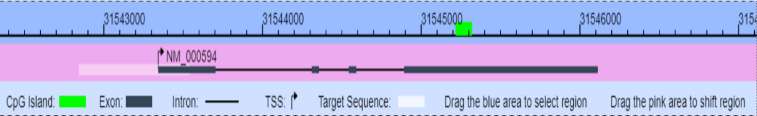  NM_000594  Genome Position: chr6 31651328-31654089(+) |
|  | 31543350 | Bottom-low vs. top-high exposure  42.15±36.60 vs.23.20±22.37  p=0.16 |  |
| *IL-4*  (16189) | Chr5: 132035956-  132036176 | Activates B-cell and T-cell proliferation induces B-cell class switching to IgE | 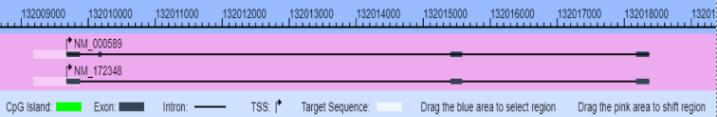  NM_000589  Genome Position: chr5 132037271-132046267(+) |
|  | 132040541  Specific primers for IL-4 gene as below:  Forward 5′-GTTGATTGGTTTTAAGTGATTGATAATT-3′ and backward 5′-Biotinylated ATACCCAAATAAATACTCACCTTTCACT-3′. | Bottom-low vs. top-high exposure  89.36±7.65 vs.85.73±6.99  p=0.258 |  |
| *MAPK1* (5594) | Chr22: 20443948- 20551970 | Mediates cell growth, adhesion, survival, and differentiation.  Regulates meiosis, mitosis and postmitotic functions | NM_002745  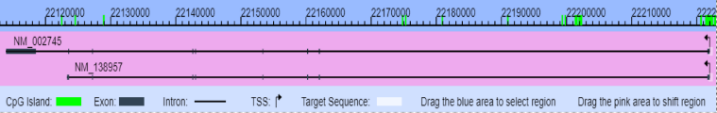Genome Position: chr22 20443948-20551970(-) |
|  | 20447613 | Bottom-low vs. top-high exposure  79.82±5.56 vs. 69.82±5.88  p=0.001 |  |
| *ESR1* (2099) | Chr6: 152128822 - 152129771 | Essential for sexual development and reproductive function, but also play a role in other tissues such as bone. | 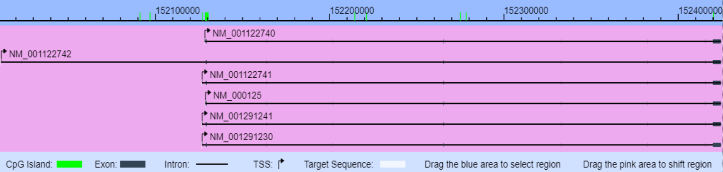  NM_000125  Genome Position: chr6: 152128813-152424408 (+) |
|  | 152128813 | Bottom-low vs. top-high exposure  0.13±0.11 vs. 0.14±0.13  p=0.86 |  |
| *ESR2*  (2100) | Chr14: 64805109 - 64805785 | Controls many cellular processes including growth, differentiation and function of the reproductive system | 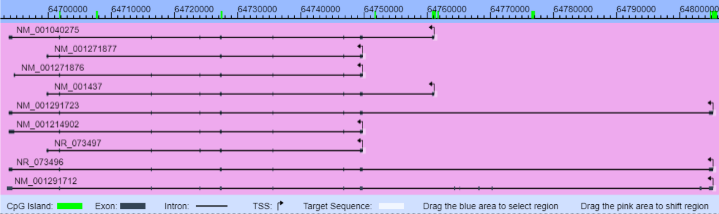  NM_001437  Genome Position: chr14: 64699746-64761128 (-) |
|  | 64805268 | Bottom-low vs. top-high exposure  0.04±0.05 vs. 0.03±0.03  p=0.75 |  |
| *PGR*  (5241) | Chr11: 100999946 - 101000437 | Plays a central role in reproductive events associated with the establishment and maintenance of pregnancy | 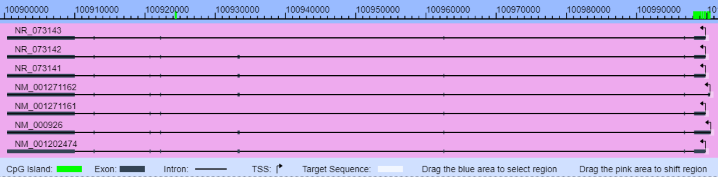  NM_001438  Genome Position: chr1: 216676587-216896814 (-)  NM_000926  Genome Position: chr11: 100900354-101000544 (-) |
|  | 101000544 | Bottom-low vs. top-high exposure  0 vs. 0 |  |
| *ESRRG*  (2104) | Chr1: 217310749 - 217311178 | Binds specifically to an estrogen response element and activates reporter genes controlled by estrogen response elements | 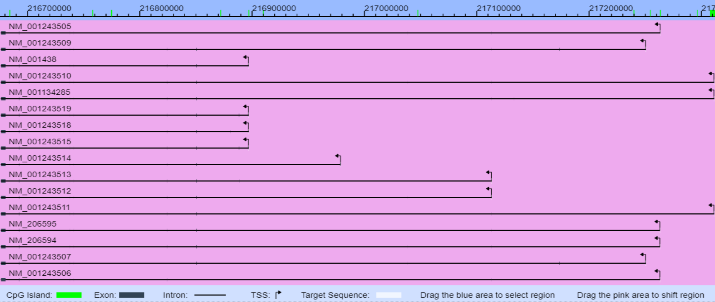 |
|  | 217311097 | Bottom-low vs. top-high exposure  0.45±0.21 vs. 0.52±0.23  p=0.491 |  |
| *THRB*  (7068) | Chr3: 24535844 – 24537436 | A nuclear hormone receptor for triiodothyronine and mediate the biological activities of thyroid hormone. | 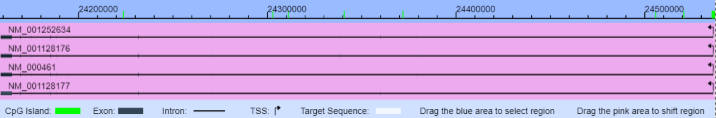  NM_000461  Genome Position: chr3: 24158644-24536313 (-) |
|  | 24536313 | Bottom-low vs. top-high exposure  10.69±23.66 vs. 15.84±27.27  p=0.642 |  |
| *CYP1A1*  (1543) | Chr15: 75018186 - 75019336 | Catalyze many reactions involved in drug metabolism and synthesis of cholesterol, steroids and other lipids. | 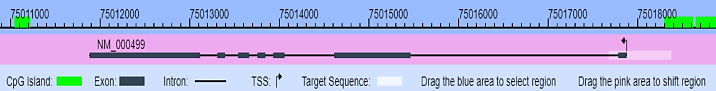  NM_000469  Genome Position: chr15: 75011882-75017877 (-) |
|  | 75017877 | Bottom-low vs. top-high exposure  15.13±25.66 vs. 4.64±15.04  p=0.26 |  |
| *CYP19A1*  (1588) | Chr15: 51633724 - 51634318 | Catalyze many reactions involved in drug metabolism and synthesis of cholesterol, steroids and other lipids. | 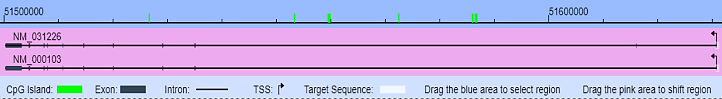  NM_000103  Genome Position: chr15: 51500253-51630795 (-) |
|  | 51630795 | Bottom-low vs. top-high exposure  100.00±0.00 vs. 100.00±0.00 |  |
| *VEGFA*  (7422) | Chr6: 43737632 – 43739852 | Induces endothelial cell proliferation, promotes cell migration, inhibits apoptosis and induces permeabilization of blood vessels. | 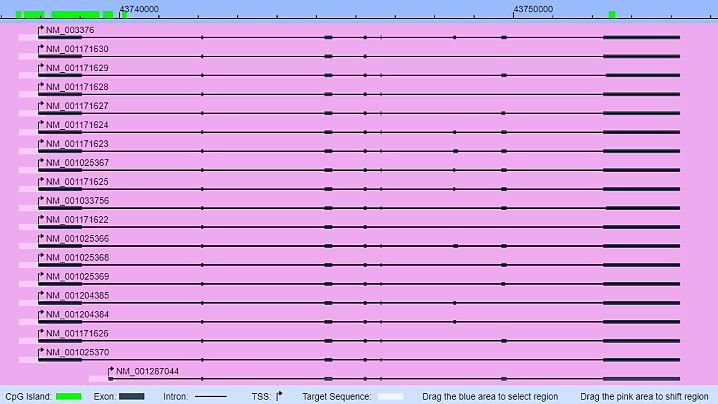  NM_001025366  Genome Position: chr6: 43737945-43754223 (+) |
|  | 43737945 | Bottom-low vs. top-high exposure  5.35±17.65 vs.4.64±15.05  p=0.92 |  |
| *MAPK3*  (5595) | Chr16: 30134220 - 30134488 | Plays also a role in initiation and regulation of meiosis, mitosis, and postmitotic functions in differentiated cells by phosphorylating a number of transcription factors | 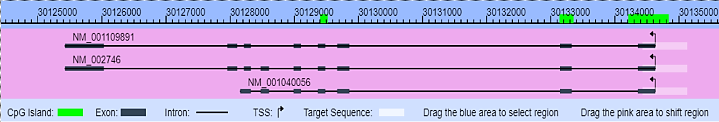  NM_001040056  Genome Position: chr16: 30128157-30134630 (-) |
|  | 30134630 | Bottom-low vs. top-high exposure  39.02±26.30 vs. 5016±26.96  p=0.37 |  |
| *STAT3*  (6774) | Chr17: 40539837 - 40540775 | Mediates cellular responses to interleukins, KITLG/SCF, LEP and other growth factors | 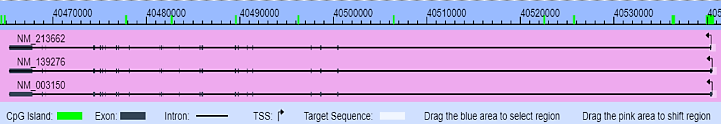  NM_003150  Genome Position: chr17: 40465342-40540513 (-) |
|  | 40540513 | Bottom-low vs. top-high exposure  0.06±0.06 vs. 0.07±0.08  p=0.77 |  |
| *LIF*  (3976) | Chr22: 30642437 - 30642671 | induction of hematopoietic differentiation in normal and myeloid leukemia cells, the induction of neuronal cell differentiation, and the stimulation of acute-phase protein synthesis in hepatocytes. | 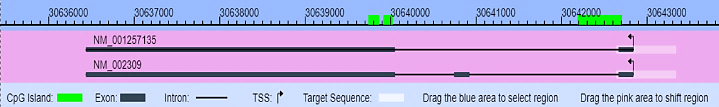  NM_002309  Genome Position: chr22: 30636435-30642840 (-) |
|  | 30642796 | Bottom-low vs. top-high exposure  0 vs. 0 |  |
| *NR1L2*  (8856) | Chr3: 119528619 -119529338 | Involved in the metabolism and secretion of potentially harmful xenobiotics, endogenous compounds and drugs. | 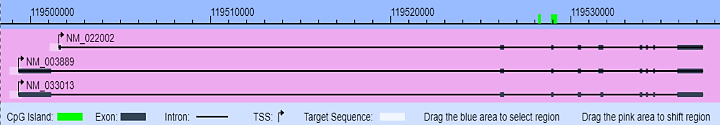  NM_003889  Genome Position: chr3: 119499330-119537332 (+) |
|  | 119501557 | Bottom-low vs. top-high exposure  0.24±0.49 vs. 0.07±0.12  p=0.287 |  |
| *TFF1*  (7031) | Chr21: 43784646 - 43785645 | Stabilizer of the mucous gel overlying the gastrointestinal mucosa that provides a physical barrier against various noxious agents | 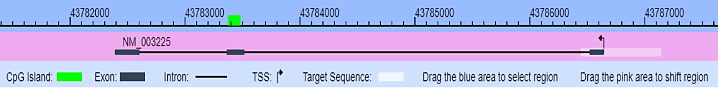  NM_003225  Genome Position: chr21: 43782390-43786644 (-) |
|  | 43786644 | Bottom-low vs. top-high exposure  99.56±0.19 vs. 99.55±0.30  p=0.934 |  |
| *S100G*  (795) | chrX:16668281- 16672793 | Belongs to a family of calcium-binding proteins that includes calmodulin, parvalbumin, troponin C, and S100 protein | 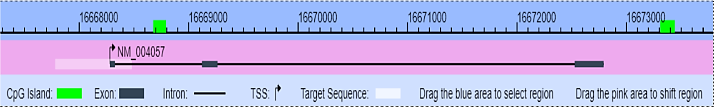  NM_004057  Genome Position: chrX 16668280-16672791(+) |
|  | 16668280 | Bottom-low vs. top-high exposure  0 vs. 0 |  |
| *LHB*  (3972) | chr19:49519237-49520347 | Encodes the beta subunit of luteinizing hormone (LH) expressed in the pituitary gland and promotes spermatogenesis and ovulation by stimulating the testes and ovaries to synthesize steroids | 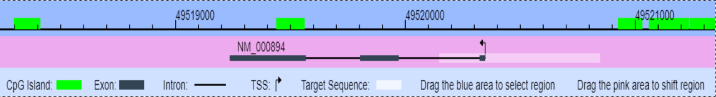  NM_000894  Genome Position: chr19 49519236-49520347(-) |
|  | 49520347 | Bottom-low vs. top-high exposure  22.56±0.13 vs. 17.55±0.30  p=0.956 |  |
| *GH1*  (2688) | chr17:61994560-61996198 | Promotes spermatogenesis and ovulation by stimulating the testes and ovaries to synthesize steroids. | 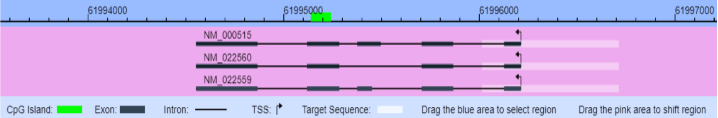  NM_000515  Genome Position: chr17:61994552-61996212 (-) |
|  | 61996212 | Bottom-low vs. top-high exposure  0.09±0.07 vs. 0.11±0.08  p=0.72 |  |
| *NR4A1*  (3164) | chr12:52416616-52453291 | Translocation of the protein from the nucleus to mitochondria induces apoptosis. | 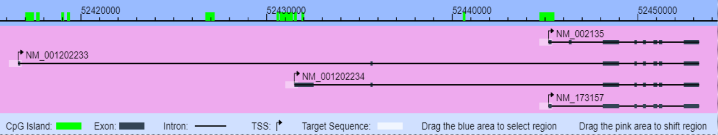  NM_001202233  Genome Position: chr12:52416615-52453291 (+) |
|  | 52416615 | Bottom-low vs. top-high exposure  4.18±14.12 vs.4.89±16.02  p=0.873 |  |
| *HOXA10*  (3206) | chr7:27210210- 27219880 | Part of the A cluster on chromosome 7 and encodes a DNA-binding transcription factor that may regulate gene expression, morphogenesis, and differentiation. | 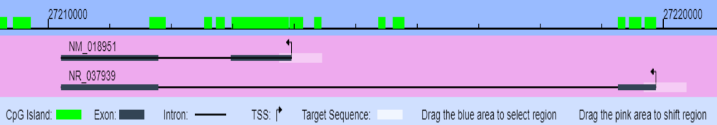  NM_037939  Genome Position: chr7:27210209-27219880 (-) |
|  | 27219880 | Bottom-low vs. top-high exposure  18.13±21.46 vs. 12.78 ±17.04  p=0.763 |  |
| *CYP11A1*  (1583) | chr15:74630100-74660081 | Catalyze many reactions involved in drug metabolism and synthesis of cholesterol, steroids and other lipids. | 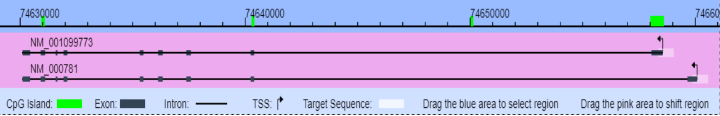  NM_000781  Genome Position: chr15:74630102-74660081 (-) |
|  | 74660081 | Bottom-low vs. top-high exposure  5.22±7.23 vs. 7.49±6.51  p=0.981 |  |
| *CYP17A1*  (1586) | chr10:104590288-104597290 | Catalyze many reactions involved in drug metabolism and synthesis of cholesterol, steroids and other lipids | 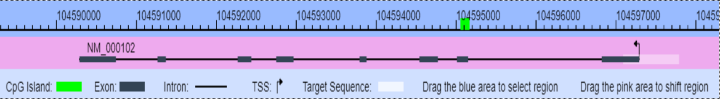  NM_000102  Genome Position: chr10:104590287-104597290 (-) |
|  | 104597290 | Bottom-low vs. top-high exposure  100.00±0.00 vs. 100.00±0.00 |  |
| *PRL*  (5617) | chr6:22287475- 22303082 | A growth regulator for many tissues, including cells of the immune system and also play a role in cell survival by suppressing apoptosis, | 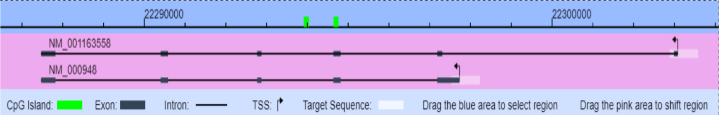  NM_001163558  Genome Position: chr6:22287472- 22303082 (-) |
|  | 22303082: | Bottom-low vs. top-high exposure  54.03±13.37 vs. 50.00±0.00  p=0.341 |  |
| *STAR*  (6770) | chr8:38000218- 38008783 | Acute regulation of steroid hormone synthesis by enhancing the conversion of cholesterol into pregnenolone | 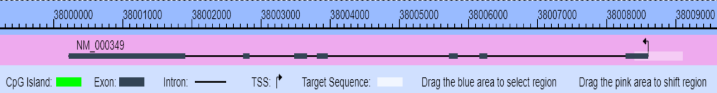  NM_000349  Genome Position: chr8:38000217- 38008600 (-) |
|  | 38008600 | Bottom-low vs. top-high exposure  25.82±3.89 vs. 26.73±4.05  p=0.598 |  |
| *IGF1*  (3479) | chr12:102789645-102874423 | Similar to insulin in function and structure and is a member of a family of proteins involved in mediating growth and development. | 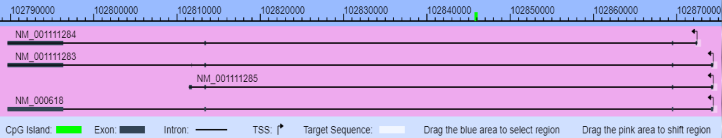  NM_001111283  Genome Position: chr12:102789644-102874378 (-) |
|  | 102874378 | Bottom-low vs. top-high exposure  38.63±3.42 vs. 37.29±4.80  p=0.665 |  |
| *NCOA1*  (8648) | chr2:24714783- 24993571 | A transcriptional coactivator for steroid and nuclear hormone receptors | 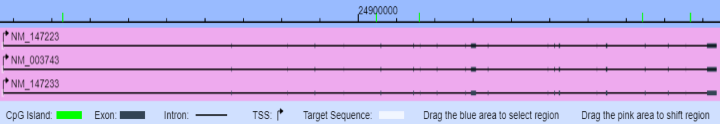  NM_147223  Genome Position: chr2:24807345- 24993570 (+) |
|  | 24807345 | Bottom-low vs. top-high exposure  0.07±0.13 vs. 0.08±0.11  p=0.81 |  |
| *P4HB*  (5034) | chr17:79801034-79818570 | As a chaperone that inhibits aggregation of misfolded proteins in a concentration-dependent manner | 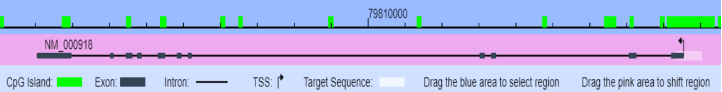  NM_000918  Genome Position: chr17:79801033-79818544 (-) |
|  | 79818544 | Bottom-low vs. top-high exposure  67.27±3.65 vs. 69.42±1.25  p=0.793 |  |
| *DDIT3*(1649) | chr12:57910371-57914300 | Implicated in adipogenesis and erythropoiesis, is activated by endoplasmic reticulum stress, and promotes apoptosis. | 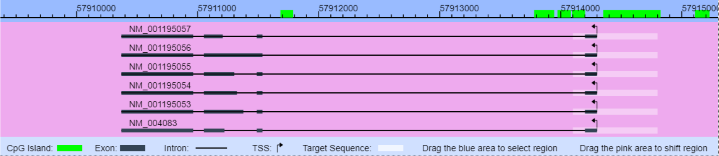  NM_001195057  Genome Position: chr12:57910370-57914300 (-) |
|  | 57914300 | Bottom-low vs. top-high exposure  31.49±5.63 vs. 32.26±4.27  p=0.52 |  |
| *FOS*  (2353) | chr14:75745477-75748937 | Implicated as regulators of cell proliferation, differentiation, and transformation. | 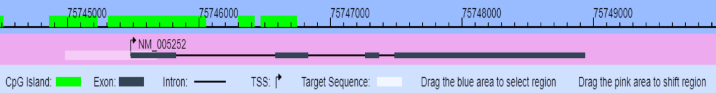  NM_005252  Genome Position: chr14:75745480-75748937 (+) |
|  | 75745480 | Bottom-low vs. top-high exposure  29.18±2.86 vs. 30.72±0.97.  p=0.66 |  |
| *HSP90AA1*  (3320) | chr14:102547075-102606086 | Aids in the proper folding of specific target proteins by use of an ATPase activity that is modulated by co-chaperones. | 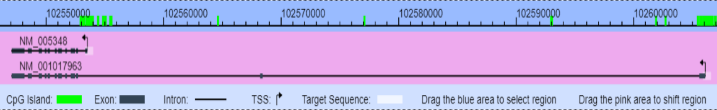  NM_001017963  Genome Position: chr14:102547074-102606086 (-) |
|  | 102606086 | Bottom-low vs. top-high exposure  77.69±3.11 vs. 76.39±5.24.  p=0.85 |  |

Figure resourse:MethPrimer 2.0
